# COVID-19 and Health Code: How Digital Platforms Tackle the Pandemic in China

**DOI:** 10.1177/2056305120947657

**Published:** 2020-08-11

**Authors:** Fan Liang

**Affiliations:** University of Michigan, USA

**Keywords:** COVID-19, Health Code, digital platforms, contact-tracing apps, health surveillance

## Abstract

As COVID-19 is rapidly spreading around the world, some countries have launched or plan to implement contact-tracing apps to detect exposure risks. In China, the government relies on Health Code, developed by Alipay and WeChat, for identifying people potentially exposed to COVID-19. The color-based code can determine people’s exposure risks and freedom of movement based on factors like travel history, duration of time spent in risky areas, and relationships to potential carriers. This essay discusses the rise of Health Code from a platform perspective, arguing that digital platforms are key players conducting health surveillance and mediating state–citizen relations in China. More importantly, tracing apps might become a normal practice in many countries, suggesting that platforms will be substantially adopted for health surveillance.

The outbreak of COVID-19 has resulted in a globally unprecedented response to health surveillance. At least 47 countries have implemented contact-tracing apps to contain the pandemic (O’Neill et al., 2020). In China, two platforms Alipay and WeChat launched Health Code, a tracing app that aims to help governments identify people potentially exposed to COVID-19 (Mozur et al., 2020). Health Code can assess people’s contagion risks based on factors like travel history, duration of time spent in risky areas, and relationships to potential carriers. The color-based code has been assigned to 900 million users in over 300 cities, determining people’s freedom of movement. The aim of this essay is to discuss how Health Code and digital platforms can mediate state–citizen relations and render citizens to a state of visibility during the spread of COVID-19.

## Code Red or Code Green

On February 9, online payment platform Alipay launched Health Code in Hangzhou. Immediately, messaging and networking platform WeChat also introduced its Health Code in China. While these two systems are installed on distinct platforms, they have similar functionalities (Mozur et al., 2020). The main purpose of Health Code is to help governments monitor and trace the transmission of COVID-19 so that cities can quickly return to normal. Using multiple data sources, Health Code can assign a color-based code to each user, determining whether people have access to a variety of activities and places. Impressively, Health Code has been applied in more than 300 Chinese cities and covers at least 900 million users.

Health Code aggregates three types of data to convert exposure risks into color-based codes (Mozur et al., 2020). First, each user needs to provide personal information, including name, national ID number, and physical conditions (e.g., fever, tiredness, dry cough). People also need to register with facial recognition and update their physical conditions every day. The second data sources are spatial-temporal data recorded by Alipay, WeChat, and other apps in daily routine usage. Geolocation data relying on smartphones’ Global Positioning System (GPS) and network carriers can determine whether users visited areas with widespread or ongoing spread, whereas temporal data can examine the duration of time spent in risky areas. Finally, Health Code adopts user networks and online transactions to evaluate whether people had contacted potential carriers of COVID-19.

**Figure 1.**
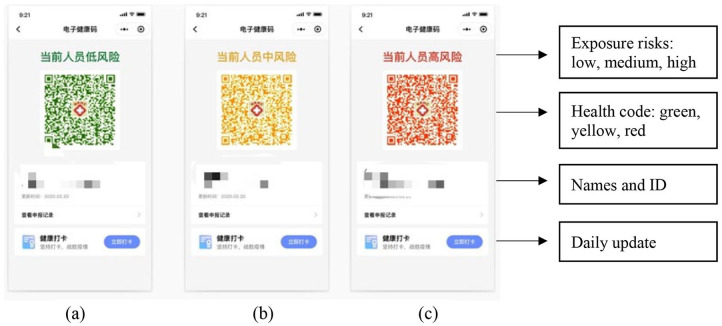
Examples of Health Code: (a) shows a green code, (b) shows a yellow code, and (c) shows a red code. Personal information and QR-codes remain anonymous.

People receive a QR-code on smartphones, indicating their exposure risks and mobility. Based on their data, all users are classified into three color-based categories: green, yellow, and red (Figure 1). While a green code suggests that the person is healthy and can move around the city freely, a yellow or red code indicates that the user has medium or high exposure risk and thus needs to self-quarantine (7–14 days). While the yellow or red code does not necessarily mean the person has the virus, it suggests that the person has a greater risk of infection. Moreover, the status of Health Code is updated at midnight daily. Thus, a user’s green code will be transformed into yellow or red if smartphone GPS data show that the user went to a risky area.

Health Code is mandatory for those who wish to visit public spaces in over 300 cities (Mozur et al., 2020). For example, people must have a green code if they travel to these cities or go to workplaces. They also need a green code in public transit, schools, airports, restaurants, hotels, and grocery stores. In some cities, people have to show their codes in local communities’ checkpoints. Those who are coded as red or yellow are restricted from visiting public spaces. Indeed, Health Code has become an essential tool to evaluate people’s mobility and everyday life in these cities.

In addition, another tech giant Baidu released its COVID-19 map, visualizing the locations and numbers of confirmed cases in 200 cities. The Chinese government also employed advanced technologies like drones, thermal cameras, and facial recognition during the outbreak of COVID-19. This suggests that governments actively leverage new technologies to tackle the pandemic in China.

## The Politics of Health Code

At the heart of the rising Health Code is the role of digital platforms. Platforms are important actors in economic and social interactions (van Dijck et al., 2018). They facilitate multisided markets and expand their services into the web (Helmond, 2015; Nieborg & Poell, 2018). In my view, digital platforms are key sociotechnical constructs mediating state–citizen relations and rendering citizens to a state of permanent visibility. In what follows, I will discuss three actors involved in Health Code: governments, digital platforms, and end users (i.e., citizens).

First and foremost, Health Code is an example of China’s recent investment in the platform ecosystem. While Health Code is installed on two commercial platforms, the underlying assumption is that governments allow tech giants to harness massive data sources. In fact, local governments have released guiding opinions and regulations to promote and normalize the use of Health Code, while Alipay and WeChat can share data with local police (Mozur et al., 2020). The State Administration for Market Regulation has issued national standards for the adoption of Health Code. Hence, state actors have become vital developers and partners of digital platforms. My previous research on China’s Social Credit System has revealed that the Chinese government is dependent upon platforms for credit assessment, suggesting the expanded cooperation between state power and private actors (Liang et al., 2018). The case of Health Code further indicates that governments and tech giants have achieved unprecedented collaborations for tracking individuals.

Second, digital platforms are pivotal gatekeepers in the data flows (Helmond, 2015). Indeed, Alipay and WeChat have penetrated deeply into the Chinese platform ecosystem and created institutional dependencies; thus, end users, advertisers, and complementors are becoming dependent upon these two platforms. The platformization of Alipay and WeChat has expanded their data collection to track and predict an ever-wider variety of users’ activities. At the same time, governments rely heavily on platforms to gather data and connect with citizens. My recent research finds that tech giant Alibaba is contributing to big data policing in China by offering technical support and data sources. Compared to the US-based platforms, Chinese platforms are important actors distributing public services and engineering power relations. For example, Alipay and WeChat provide electronic ID for individual users, so people can access government services without traditional state-issued ID cards. Thus, Health Code shows that Alipay and WeChat can mediate state–citizen relations by capturing personal data for public health surveillance.

Finally, we need to think about the datafication of individuals. Big data and computational revolutions have substantially promoted surveillance and social sorting (Cheney-Lippold, 2018). The proliferation of the sharing economy seizes the opportunity to constantly classify individuals. Recently, China deploys an official platform (i.e., *Xuexi Qiangguo*) to rate and rank citizens based on multiple metrics, including how many news articles people read and how many correct answers they provide in quizzes. Users receive study points and national rankings, further quantifying their political knowledge and loyalty. Health Code can be considered as a rating and ranking practice aiming to render citizens to a state of visibility. Consequently, citizens are increasingly becoming visible and governable through the intersection of platform datafication and everyday life.

It is worth noting that Health Code raises concerns about accuracy, privacy, and security. Currently, the system is largely inconsistent at local levels. For instance, a yellow code indicates 7 days quarantine in Hangzhou, while the same color means 14 days in Shandong province. Meanwhile, a green code does not necessarily mean the person is healthy, since several people with green codes tested positive for COVID-19 in Wuhan. More importantly, Health Code poses a threat to data security and reflects tensions between public values and privacy. It is not clear, for instance, who is in control of data flows, who owns user health data, and how Health Code is regulated by governments.

## A New Model in Post-COVID-19 Life?

In this essay, I am not suggesting that “what happens in China, stays in China.” In fact, it is misleading to consider the response to COVID-19 as a distinction between autocracies and democracies (Ang, 2020). I have come to believe that China’s Health Code is not new, since we have seen the emergence of rating technology and predictive algorithms on Google and Facebook. More recently, public and private actors in 47 countries have implemented contact-tracing apps, such as Australian COVIDSafe, French StopCovid, and Mexican CovidRadar (O’Neill et al., 2020). While most tracing apps are voluntary (e.g., France, Germany, and Malaysia), people are compelled to use these tools in India, Qatar, and Turkey. Meanwhile, Apple and Google are jointly developing tracing technology in 23 countries. Therefore, an important question to ask is as follows: will China’s Health Code be a new model in post-COVID-19 life?

We find ourselves in a dilemma: the choice between controlling COVID-19 and resuming public life. In the foreseeable future, we will see more tracing tools around the world and hear more debates about public values and privacy. In the United States, state governments and tech firms have launched multiple tracing apps (e.g., Covid Watch and NOVID). North Dakota, for example, released an app called Care19 before it reopened on May 1. As such, it is possible that tracing apps will normalize health surveillance and become a part of daily life in many countries. Of course, I am not saying that Health Code will become the standard for other countries. Indeed, the application of tracing apps relies on many factors like transparency, trust in government, and citizens’ cooperation. It is too early to be concluding on the effects of Health Code and other tracing apps. But it is clear that these technologies will have a long-term impact on our everyday life.
